# Calcium requirements for Japanese quail in the early growth period

**DOI:** 10.1016/j.psj.2025.105209

**Published:** 2025-04-25

**Authors:** Mostafa Ahmadi, Kamal Shojaeian, Mahmoud Ghazaghi, Mehran Mehri

**Affiliations:** Department of Animal Sciences, Faculty of Agriculture, University of Zabol; Sistan, 98661-5538, Iran

**Keywords:** Bone development, Broken-line, Calcium, Immunity, Japanese quail

## Abstract

This study aimed to determine the optimal dietary calcium (Ca) requirement for quail chicks by evaluating its effects on growth performance, carcass characteristics, serum biochemical parameters, immune responses, and bone development. Quail chicks were fed diets containing varying levels of Ca (0.60–1.00 %) in a completely randomized design from 11 to 24 days posthatch. Growth performance data indicated that weight gain was influenced by dietary Ca (*P* = 0.006), with the highest gain observed at 0.80 % Ca. Feed conversion ratio (FCR) tended to follow a quadratic trend (*P* = 0.092), with optimal efficiency estimated at 0.80 % Ca. Carcass analysis showed that thigh meat yield (TMY) was affected by dietary Ca levels (*P* = 0.012), whereas breast meat yield (BMY) and overall carcass yield remained unaffected. Serum biochemical parameters, including Ca, phosphorus, alkaline phosphatase (ALP), and insulin-like growth factor (IGF), did not show differences among dietary treatments (*P* > 0.05). Immune responses were not markedly affected, although total immunoglobulins (Ig-Total) and IgY exhibited slight improvements at 0.80 % Ca. Bone parameters, including bone diameter and thickness, demonstrated robust responses to dietary Ca, with the highest R² values observed at approximately 0.80 % Ca. Two-slope broken-line regression models estimated the Ca requirement for optimal gain and FCR at 0.788 % and 0.800 %, respectively. The best estimate for BMY and TMY requirements was 0.809 % and 0.748 %, respectively, while bone density suggested an optimal requirement of 0.835 %. Immune responses indicated a higher Ca requirement of approximately 0.852 %, which was greater than the requirements for performance and other physiological traits. Based on the combined highest R² and lowest standard deviation of residuals (*S_y.x_*), the overall optimal dietary Ca requirement for quail chicks was estimated at 0.800 % for growth and efficiency, but higher levels may be needed to support bone density and immune function. These findings provide valuable insights for optimizing Ca nutrition in quail production.

## Introduction

Calcium plays a crucial role in the development and health of growing Japanese quail (Coturnix japonica), impacting bone formation, muscle function, and overall physiological processes (Perine et al., 2022; Pourmollaei et al., 2024). As the economic importance of quail for meat production increases, understanding their specific nutritional needs becomes paramount for optimizing their health and productivity (Pourmollaei et al., 2024). Recent research has highlighted the need for more precise Ca requirements for growing quail, as previous studies have often relied on generalized poultry guidelines that may not be suitable for this species (Mariz et al., 2017; Pourmollaei et al., 2024). Inadequate Ca intake leads to poor bone mineralization, increased fracture risk, and negatively impact growth and production efficiency (Perine et al., 2022; Pourmollaei et al., 2024).

Studies have shown that Ca requirements for growing Japanese quail may vary depending on age and production stage. For quail between 15 and 35 days old, Ca requirements for maximum growth and performance have been estimated to be greater than or equal to 0.90 %1. However, other research suggests that growing quail may require no more than 0.5 % Ca in their diet (Edache et al., 2005; Reddy et al., 1980). The discrepancies in reported Ca requirements highlight the importance of using appropriate statistical models and methodologies to accurately determine nutrient needs (Pourmollaei et al., 2024). Some studies have employed quadratic models to estimate optimal Ca levels, with results indicating that dietary Ca between 0.75 % and 0.87 % may be optimal for growth performance and bone health in quail during the post-hatch growth period (Pourmollaei et al., 2024).

It is worth noting that Ca requirements for optimal bone density may exceed those for growth performance, suggesting that dietary recommendations should consider both factors (Pourmollaei et al., 2024). Additionally, the interplay between Ca and other nutrients, such as phosphorus, should be taken into account when formulating diets for growing quail (Mariz et al., 2017; Reddy et al., 1980). Further research using robust modeling techniques and considering various response variables is necessary to establish more accurate and specific Ca requirements for growing Japanese quail. This will enable the optimization of feed formulation, leading to improved productivity and welfare in commercial quail production settings (Mariz et al., 2017; Pourmollaei et al., 2024).

Although Ca and phosphorus (P) exhibit well-documented interactive effects on mineral metabolism and bone physiology (Wu et al., 2023), the present study aimed to determine the optimal dietary Ca requirement under a fixed, practical level of non-phytate P using an univariate model. While factorial designs are valuable for characterizing nutrient interactions, univariate regression remains a scientifically valid and widely employed method for estimating nutrient requirements, including those reported in NRC guidelines. This research investigates the Ca requirements of Japanese quail during the second and third weeks posthatch, using growth performance, carcass attributes, tibia characteristics, serum biochemical markers, and immunological response.

## Materials and methods

### Ethics statement

This study protocol adheres to the guidelines established by the Iranian Council of Animal Care and ARRIVE/NIH (Du Sert et al., 2020), and it has been approved by the Research Animal Ethics Committee (AECUZ-2012-BR) at the University of Zabol.

### Chemical analysis

Analysis of the feed ingredient samples was conducted to determine the dry matter (DM; method 930.15, AOAC, 2006), ash content (method 942.05, AOAC, 2006), crude fiber (method 978.10, AOAC, 2006), ether extract (method 2003.05, AOAC, 2006), Ca (method 934.01,AOAC (2006), phosphorus (method 965.17,AOAC, 2006), and crude protein (CP; method 990.03, AOAC, 2006).

### Birds and experimental diets

To estimate Ca requirements, a dose-response experiment was performed on Japanese quail from 11 to 24 days of age. One-day-old chicks were obtained from the experimental farm at the University of Zabol, Iran. According to NRC (1994), a standard diet meeting the nutritional needs of growing Japanese quails was provided from hatch day to day 10. A basal quail diet (meeting all nutritional needs except Ca; see Table 1) was formulated, then five experimental diets with 0.60 % to 1.00 % Ca (in 0.10 % increments) were developed. A total of 450 birds, weighed on day 11, were randomly divided into 5 experimental treatments; each treatment had 6 floor pens of 15 birds. The animals had free access to food and water during the experiment. Room temperature was kept at 27 ± 2.5 ºC, and humidity at 60 ± 3.3 %. An 18L:6D photoperiod was maintained throughout the experiment.

**Table 1:** Composition of basal diet.

Ingredient	Amount (%)
Corn, grain	53.21
Soybean Meal (44 %)	35.00
Corn gluten meal (60 %)	5.31
Sand	2.03
Soybean oil	1.00
Di-calcium phosphate	0.89
Limestone	0.78
NaHCO_3_	0.33
DL-Methionine	0.33
NaCl	0.29
Mineral Premix^1^	0.25
Vitamin Premix^2^	0.25
L-Lysine HCl	0.23
L-Threonine	0.10
Nutrient composition	
AME (Kcal/kg)	2920
CP (%)	25.0
Total Lysine (%)	1.38
Total Methionine + Cysteine (%)	1.11
Total Methionine (%)	0.72
Total Tryptophan (%)	0.27
Total Arginine (%)	1.54
Total Threonine (%)	1.02
Ca (%)	0.60
P available (%)	0.35
Na (%)	0.23
Cl (%)	0.26
K (%)	0.88
DEB^3^ (mEq/kg)	250

^a^Mineral premix provided per kilogram of diet: Mn (from MnSO4·H2O), 65 mg; Zn (from ZnO), 55 mg; Fe (from FeSO4·7H2O), 50 mg; Cu (from CuSO4·5H2O), 8 mg; I [from Ca (IO3)2·H2O], 1.8 mg; Se, 0.30 mg; Co (from Co2O3), 0.20 mg; Mo, 0.16 mg.

^b^Vitamin premix provided per kilogram of diet: vitamin A (from vitamin A acetate), 11,500 IU; cholecalciferol, 2,100 IU; vitamin E (from dl-α-tocopheryl acetate), 22 IU; vitamin B12, 0.60 mg; riboflavin, 4.4 mg; nicotinamide, 40 mg; calcium pantothenate, 35 mg; menadione (from menadione dimethylpyrimidinol), 1.50 mg; folic acid, 0.80 mg; thiamine, 3 mg; pyridoxine, 10 mg; biotin, 1 mg; choline chloride, 560 mg; ethoxyquin, 125 mg.

^c^Dietary Electrolyte Balance: represents dietary Na +*K* − Cl in mEq/kg of diet.

### Performance and carcass attributes

Cage-basis body weight, weight gain, and feed intake were recorded from d 11 to 24, allowing for feed conversion ratio calculation. The study did not reveal any instances of mortality. At the end of the experimental period, quail chicks were individually weighed using a digital scale with a precision of 0.01 g. After recording live body weight, birds were euthanized using CO_2_ asphyxiation and the relative weight of carcass, breast meat yield (BMY), thigh meat yield (TMY), and internal organs, including the bursa, liver, heart, spleen, and pancreas, was determined as a percentage of live body weight.

### Tibia characteristics

At the conclusion of the trial, two birds from each replicate were humanely sacrificed for skeletal analysis. Both tibiae were excised, stripped of adhering tissues (muscles, fat, tendons), and immediately placed in labeled plastic bags, then stored at –20°C pending further evaluation. Prior to analysis, bones were boiled and meticulously cleared of residual soft tissue. They were then weighed, oven-dried at 109°C for 12 hours, immersed in acetone for 48 hours to remove lipids, re-dried under the same conditions, and subsequently incinerated in a muffle furnace at 550°C overnight to determine bone ash content, according to the protocol of Asensio et al. (2020). Bone dimensions and mass were determined using a precision digital caliper and electronic scale, respectively (Pourmollaei et al., 2024). The Seedor index, a measure of bone density, was calculated as the ratio of tibial weight to length (Seedor et al., 1991).

### Serum biochemical parameters

Sterile syringes collected blood samples from the wing vein of quails on day 35. Serum Ca, P, alkaline phosphatase (ALP), and insulin like growth factor (IGF) were measured using a Pars-Azmoun (Tehran, Iran) commercial kit after a 30-minute room temperature blood clot and a 10-minute centrifugation at 3,000 rpm.

### Immunoglobulin assessment

In each replicate, four birds were administered sheep red blood cell (SRBC) antigens on days 15 and 20. Following the second antigen challenge, antibody production against SRBC was quantified via hemagglutination inhibition testing of serum samples, as per the methodology of Cheema et al. (2003). Blood samples were collected from the jugular vein of quail using sterile syringes. The collected blood was allowed to clot at room temperature for 30 minutes, followed by centrifugation at 3,000 × *g* for 10 minutes to separate the serum. The obtained serum samples were stored at −20°C until immunoglobulin analysis. Serum IgY, IgM, and total immunoglobulin (Ig-Total) concentrations were determined using enzyme-linked immunosorbent assay (ELISA) kits specific to avian species, following the manufacturer’s protocols (Calisher et al., 1986; Esmailnejad et al., 2019). The absorbance was measured at 450 nm using a microplate reader (Immunoskan BDSL, Thermo Lab. Systems, Finland). The standard curve was generated using serial dilutions of purified immunoglobulins, and sample concentrations were extrapolated accordingly.

### Statistical analysis

The data were analyzed using the GLM procedure in SAS (2002) to assess the linear and quadratic responses of birds to dietary Ca, considering the average value of each pen as the experimental unit. Calcium requirements were estimated using various broken-line regression models, following the methodology of Khosravi et al. (2016), as outlined below:

Two-slope broken line:

Linear ascending (or descending)-linear descending (or ascending):

Y=L+U×(R−−X)×(X<R)+V×(X−−R)×(X>R)

Quadratic ascending (or descending)-linear descending (or ascending):

$$Y=L+U\times {\left(R--X\right)}^{2}\times \left(X<R\right)+V\times \left(X--R\right)\times \left(X>R\right)$$

Quadratic ascending (or descending)-quadratic descending (or ascending):

$$Y=L+U\times {\left(R--X\right)}^{2}\times \left(X<R\right)+V\times {\left(X--R\right)}^{2}\times \left(X>R\right)$$

where Y represents the bird's response; L denotes the asymptote for the first segment; U and V correspond to the slopes of the first and second segments, respectively, indicating an increasing or decreasing trend; and R represents the breakpoint, which is considered the Ca requirement.

Model selection for each variable prioritized the highest R², lowest SE, and lowest *S*_y_.ₓ (residual standard deviation):

$$S_{y.x}=\;\sqrt{\frac{SS}{df}}$$

## Results

### Growth performance

As indicated in Table 2, dietary Ca level significantly influenced gain (*P* = 0.006) and FCR (*P* = 0.037) in quail chicks, while feed intake remained statistically unaffected by dietary calcium (*P* = 0. 976). The highest gain (7.27 g) was observed at 0.80 % Ca, showing a quadratic response (*P* = 0.005), whereas both lower and higher levels resulted in reduced gains. The FCR demonstrated a quadratic trend (*P* = 0.017), decreasing at 0.80 % Ca (FCR = 3.58), but increased at 0.90 % and 1.00 %.

**Table 2:** Growth of quail chicks fed different amounts of dietary calcium.

Response	Dietary calcium (%)					SEM	Probabilities		
	0.60	0.70	0.80	0.90	1.00		Model	Linear	Quadratic
Feed intake (g)	26.0	26.5	25.9	26.3	26.0	0.81	0.976	0.906	0.835
Gain (g)	6.80	7.13	7.27	6.27	6.00	0.24	0.006	0.005	0.019
FCR^1^	3.82	3.77	3.58	4.21	4.34	0.18	0.037	0.017	0.092

1 Feed conversion ratio.

### Carcass characteristics

Relative weights of carcass components, including BMY and TMY, were affected by dietary Ca (Table 3). BMY and TMY exhibited quadratic responses (*P* = 0.030 and *P* = 0.015, respectively), with peak values at 0.80 % calcium. Dressing percent tended to decrease linearly with increasing Ca levels (*P* = 0.061). Other internal organs such as liver (*P* = 0.172), heart (*P* = 0.794), pancreas (*P* = 0.905), spleen (*P* = 0.468), and bursa (*P* = 0.594) were not influenced by Ca level.

**Table 3:** Relative weight of carcass attributes (%) of quail chicks fed different levels of dietary calcium.

Response	Dietary calcium (%)					SEM	Probabilities		
	0.60	0.70	0.80	0.90	1.00		Model	Linear	Quadratic
Carcass	61.3	61.6	61.6	60.8	59.3	0.80	0.222	0.061	0.142
BMY^1^	26.9	27.6	28.0	27.8	27.4	0.38	0.332	0.331	0.061
TMY^2^	14.1	14.5	14.5	13.9	13.8	0.18	0.012	0.030	0.015
Bursa	0.12	0.14	0.15	0.12	0.10	0.02	0.594	0.461	0.171
Liver	2.53	2.70	2.61	2.80	2.65	0.08	0.172	0.174	0.248
Heart	0.79	0.79	0.75	0.78	0.78	0.02	0.794	0.793	0.464
Spleen	0.43	0.10	0.34	0.09	0.10	0.17	0.468	0.224	0.752
Pancreas	0.40	0.43	0.41	0.42	0.45	0.04	0.905	0.426	0.882

1 Breast meat yield.

2 Thigh meat yield.

### Tibia characteristics

Tibia parameters responded variably to calcium levels (Table 4). Tibia weight increased with calcium (*P* = 0.004), peaking at 0.80 % calcium. The Seedor index showed a quadratic response (*P* = 0.005), reaching the maximum (19.0 mg/mm) at 0.80 %. However, tibia length (*P* = 0.326) and ash content (*P* = 0.438) were not different among treatments.

**Table 4:** Tibia characteristics of quail chicks fed different levels of dietary calcium.

Response	Dietary calcium (%)					SEM	Probabilities		
	0.60	0.70	0.80	0.90	1.00		Model	Linear	Quadratic
Tibia weight (g)	0.60	0.66	0.70	0.66	0.59	0.02	0.004	0.825	0.001
Tibia length (mm)	35.7	36.4	36.6	36.9	35.9	0.43	0.326	0.513	0.057
Seedor index (mg/mm)	16.8	18.3	19.0	18.0	16.5	0.65	0.063	0.689	0.005
Tibia ash (%)	44.1	44.6	43.7	43.5	42.1	1.06	0.438	0.133	0.450

### Serum biochemical parameters

As shown in Table 5, serum Ca (*P* = 0.883), P (*P* = 0.241), and ALP (*P* = 0.947) concentrations were not affected by dietary Ca levels. However, IGF levels showed a quadratic tendency (*P* = 0.062), with the highest concentration (4.20 µg/mL) recorded at 0.80 % Ca.

**Table 5:** Serum concentration of calcium (Ca), phosphorus (P), alkaline phosphatase (ALP), and insulin-like growth factor (IGF) of quail chicks fed different levels of dietary calcium.

Response	Dietary calcium (%)					SEM	Probabilities		
	0.60	0.70	0.80	0.90	1.00		Model	Linear	Quadratic
Ca (mg/dL)	9.24	9.40	9.62	9.36	9.27	0.28	0.883	0.982	0.353
P (mg/dL)	5.99	6.77	6.41	6.31	6.28	0.24	0.241	0.889	0.129
ALP (U/L)	1965	2032	2054	1902	1832	217	0.947	0.568	0.585
IGF (µg/mL)	3.08	3.43	4.20	3.55	3.40	0.33	0.220	0.480	0.062

### Immune responses

Antibody titers against SRBC antigen were moderately influenced by dietary Ca (Table 6). Ig-Total (*P* = 0.110) and IgY (*P* = 0.044) titers quadratically responded to increasing Ca in diet, with peak values at 0.90 % and 0.80 % Ca, respectively. IgM was not affected by calcium level (*P* = 0.456).

**Table 6:** Antibody production (total immunoglobulins: Ig-Total; IgY, and IgM) against sheep red blood cell (SRBC)-antigen in quail chicks fed different levels of dietary calcium.

Response	Dietary calcium (%)					SEM	Probabilities		
	0.60	0.70	0.80	0.90	1.00		Model	Linear	Quadratic
Ig-Total (log_2_)	2.30	2.70	2.90	3.00	2.70	0.25	0.328	0.164	0.110
lgY (log_2_)	1.20	1.50	1.60	1.50	1.40	0.18	0.234	0.486	0.044
lgM(log_2_)	1.10	1.20	1.30	1.50	1.30	0.15	0.456	0.203	0.281

### Estimation of calcium requirements

Broken-line modeling indicated that the Ca requirement for optimal gain and FCR ranged from 0.638 to 0.788 % and 0.709 to 0.80 %, respectively, based on different ascending and descending segments (Table 7). The best estimations for gain and FCR based on maximum R² and minimum *S*_y.x_ were 0.788 and 0.80 %, respectively.

**Table 7:** Estimation of calcium requirements for performance of quail chicks using two-slope broken line models.

Response	Estimate		Accuracy	
Gain (g)	Requirement	SD^1^	R^2^	*S*_y.x_^2^
Two-slope linear-ascending linear-descending	0.768	0.065	0.926	0.2980
Two-slope quadratic-ascending linear-descending	**0.788**	0.063	0.926	0.2980
Two-slope quadratic-ascending quadratic-descending	0.636	0.446	0.854	0.4181
Feed conversion ratio				
Two-slope linear-ascending linear-descending	**0.800**	0.114	0.900	0.1999
Two-slope quadratic-ascending linear-descending	0.800	0.085	0.895	0.2047
Two-slope quadratic-ascending quadratic-descending	0.709	0.258	0.792	0.2884

1 Standard deviation.

2 Standard deviation of the residuals.

Broken-line modeling indicated that the Ca requirement for optimal BMY and TMY ranged from 0.809 to 0.847 % and 0.748 to 0.773 %, respectively, depending on the ascending and descending segments applied (Table 8). The best estimations for BMY and TMY based on maximum R² and minimum *S*_y.x_ were 0.809 % and 0.773 %, respectively.

**Table 8:** Estimation of calcium requirements for breast meat yield (BMY) and thigh meat yield (TMY) of quail chicks using two-slope broken line models.

Response	Estimate		Accuracy	
BMY (%)	Requirement	SD^1^	R^2^	*S*_y.x_^2^
Two-slope linear-ascending linear-descending	**0.809**	0.018	0.993	0.0694
Two-slope quadratic-ascending linear-descending	0.847	0.026	0.989	0.0843
Two-slope quadratic-ascending quadratic-descending	0.823	0.050	0.976	0.1276
TMY (%)				
Two-slope linear-ascending linear-descending	**0.748**	0.049	0.923	0.1837
Two-slope quadratic-ascending linear-descending	0.773	0.057	0.923	0.1837

1 Standard deviation.

2 Standard deviation of the residuals.

Broken-line modeling estimated the Ca requirement for optimal Seedor index to range from 0.777 to 0.835 % across different models (Table 9). The best estimation was 0.835 %, as derived from the quadratic-ascending linear-descending model, which provided the highest R² (0.999) and lowest *S*_y.x_ (0.0418), indicating exceptional model precision.

**Table 9:** Estimation of calcium requirements for Seedor index (mg/mm) of tibia in quail chicks using two-slope broken line models.

Response	Estimate		Accuracy	
Seedor index	Requirement	SD^1^	R^2^	*S*_y.x_^2^
Two-slope linear-ascending linear-descending	0.777	0.018	0.989	0.2123
Two-slope quadratic-ascending linear-descending	**0.835**	0.004	0.999	0.0418
Two-slope quadratic-ascending quadratic-descending	0.789	0.035	0.984	0.2619

1 Standard deviation.

2 Standard deviation of the residuals.

Broken-line models estimated that the Ca requirement for IgY, Ig-Total, and bursa development ranged from 0.750 to 0.80 %, 0.842 to 0.879 %, and 0.774 to 0.80 %, respectively, depending on model structure (Table 10). The best estimates based on highest R² and lowest *S*_y.x_ were 0.80 % for IgY, 0.852 % for Ig-Total, and 0.774 % for bursa, indicating the important role of Ca in supporting immunocompetence in quail chicks.

**Table 10:** Estimation of calcium requirements for immune parameters of quail chicks using two-slope broken line models.

Response	Estimate		Accuracy	
IgY (log_2_)	Requirement	SD^1^	R^2^	*S*_y.x_^2^
Two-slope linear-ascending linear-descending	0.750	-	1.00	-
Two-slope quadratic-ascending linear-descending	**0.800**	-	1.00	-
Two-slope quadratic-ascending quadratic-descending	0.778	0.031	0.988	0.0336
Ig-Total (log_2_)				
Two-slope linear-ascending linear-descending	0.842	0.056	0.932	0.2041
Two-slope quadratic-ascending linear-descending	0.879	0.033	0.983	0.1022
Two-slope quadratic-ascending quadratic-descending	**0.852**	0.042	0.987	0.0891
Bursa (%)				
Two-slope linear-ascending linear-descending	**0.774**	0.020	0.989	0.0041
Two-slope quadratic-ascending linear-descending	0.800	0.033	0.988	0.0042
Two-slope quadratic-ascending quadratic-descending	0.744	0.089	0.921	0.1097

1 Standard deviation.

2 Standard deviation of the residuals.

Calcium requirements for optimizing ALP and IGF responses ranged from 0.688 to 0.791 % and 0.80 to 0.825 %, respectively (Table 11). The best estimations based on the highest R² and lowest *S*_y.x_ values were 0.791 % for ALP and 0.80 % for IGF, suggesting these biomarkers are responsive to moderate elevations in dietary Ca.

**Table 11:** Estimation of calcium requirements for physiological parameters (alkaline phosphatase: ALP; insulin-like growth factor: IGF) of quail chicks using two-slope broken line models.

Response	Estimate		Accuracy	
ALP (U/L)	Requirement	SD^1^	R^2^	*S*_y.x_^2^
Two-slope linear-ascending linear-descending	0.768	0.040	0.967	33.68
Two-slope quadratic-ascending linear-descending	**0.791**	0.039	0.967	33.68
Two-slope quadratic-ascending quadratic-descending	0.688	0.209	0.902	57.65
IGF (mg/L)				
Two-slope linear-ascending linear-descending	**0.800**	0.069	0.909	0.2488
Two-slope quadratic-ascending linear-descending	0.825	0.142	0.781	0.3857
Two-slope quadratic-ascending quadratic-descending	0.811	0.189	0.705	0.4473

1 Standard deviation.

2 Standard deviation of the residuals.

### Summary of estimated calcium requirements

An integrated analysis of all responses indicated that the optimal Ca levels for different physiological and performance parameters ranged between 0.748 % and 0.852 %. The most precise estimates were for the Seedor index (0.835 %, CI: 0.827–0.843), which was higher than that recommended by NRC (1994).

## Discussion

This study investigated the optimal Ca requirements for growing Japanese quail, examining various parameters related to growth, carcass characteristics, tibia density, serum biochemical markers, and immune function. The findings reveal specific Ca needs for different physiological aspects in quail chicks, providing valuable insights for optimizing nutritional strategies in quail production. The results demonstrate that dietary Ca levels influence growth performance in growing quail chicks, with the optimal level for gain determined to be 0.788 % and FCR optimized at 0.80 % of diet. The quadratic response observed in growth parameters suggests that both insufficient and excessive Ca levels can negatively impact productive performance in quail. These findings align closely with a recent study by Pourmollaei et al. (2024) that identified dietary Ca requirements between 0.75 % and 0.84 % for gain and 0.74 % to 0.83 % for FCR in quail during two last weeks of rearing period. These researchers showed that Ca requirements for bone density and strength may be higher than that needs for optimal performance at the end of growth period.

It is noteworthy that feed intake remained consistent across treatments despite varying Ca levels. This suggests that Ca concentration within the range tested does not significantly influence palatability or feed consumption patterns in growing quail. Pourmollaei et al. (2024) similarly observed that gain increased quadratically with increasing dietary Ca levels, while FCR showed a tendency to decrease, indicating improved efficiency. This pattern of response indicates that Ca primarily affects nutrient utilization rather than intake when provided within reasonable dietary ranges. The optimal Ca level of approximately 0.80 % identified in our study for growth performance corresponds with historical recommendations, but differs from earlier research. Miller (1967) reported satisfactory weight gain in growing Japanese quails with just 0.44 % Ca, while our studies demonstrated that modern strains required approximately 0.82 % Ca for maximum weight gain (Pourmollaei et al., 2024). This significant difference highlights the impact of genetic selection on nutritional requirements over time, emphasizing the need for updated guidelines for contemporary quail strains.

The analysis of carcass attributes revealed interesting patterns, with TMY showing a significant quadratic response to dietary Ca levels, peaking at 0.80 % Ca. The optimal Ca requirement for TMY was estimated at 0.748 %, slightly lower than the requirement for growth parameters. In contrast, BMY and overall carcass yield showed no significant differences across treatments, though the estimated optimal Ca requirement for BMY was 0.809 %, suggesting a slight tissue-specific response to Ca supplementation in growing quail. The differential response between TMY and BMY may be attributed to the physiological and biochemical differences between these muscle types. Thigh muscles, primarily composed of slow-twitch fibers, exhibit higher mitochondrial content and enhanced oxidative metabolism compared to breast muscles. Research indicates that slow-twitch fibers are more abundant in thigh muscles, which correlates with increased mitochondrial density and oxidative capacity, facilitating sustained aerobic activity (Crowther and Gronka, 2002; Stuart et al., 2013). In contrast, breast muscles, which contain a higher proportion of fast-twitch fibers, are adapted for short bursts of power and have lower mitochondrial content (Reverter et al., 2017). The literature on the role of Ca in muscle development in poultry indicates that Ca is essential for muscle contraction and function, potentially explaining the observed tissue-specific responses (Hofmann et al., 2021). While Pourmollaei et al. (2024) focused primarily on bone parameters rather than meat yield, their findings on the influence of Ca on overall growth indirectly support our observations of muscle development.

The lack of effect on organ weights, including liver, heart, spleen, and pancreas, indicates that dietary Ca levels within the tested range do not substantially influence visceral organ development in growing quail. This observation aligns with previous research suggesting that Ca primarily affects skeletal development and muscle growth, rather than visceral organ development in poultry species (El-Katcha et al., 2015; Garcia et al., 2024).

Serum biochemical parameters, including Ca and P concentrations, showed no significant differences across dietary treatments. This finding is consistent with the homeostatic mechanisms that maintain blood Ca levels within a narrow physiological range, regardless of dietary intake, if minimum requirements are met. Similarly, it has been reported that Ca, P, and ALP levels in blood serum were not significantly influenced by available P levels in broiler chickens (Imari et al., 2020), indicating that serum biochemical parameters may not be sensitive indicators of dietary mineral status within certain ranges. The absence of consistent trends in ALP and IGF concentrations related to Ca levels suggests complex physiological interactions. Nevertheless, the estimated Ca requirement for optimal ALP activity was 0.791 %, close to the requirement for growth parameters, providing internal consistency to our findings. Recent research indicates that the relationship between dietary Ca and physiological responses is complex and influenced by various factors. Excessive dietary Ca can negatively impact P digestibility by forming insoluble complexes with phytate at high pH levels, which reduces the availability of both minerals for absorption (David et al., 2023; Li et al., 2021). This interaction is particularly significant in poultry nutrition, where the balance of Ca and P is crucial for optimal growth and bone health (Schlegel and Gutzwiller, 2020), therefore, careful management of dietary Ca levels is necessary to ensure optimal P utilization and overall health in poultry.

The present study indicates a relatively high dietary Ca requirement for Japanese quail during the early growth phase (second and third weeks of age), with an optimal Seedor index estimated at 0.835 % Ca based on the quadratic-ascending linear-descending model. This estimate is close to the 0.86 % Ca requirement reported by Pourmollaei et al. (2024) for older quail (fourth and fifth weeks of age), suggesting that younger birds may also require high dietary Ca concentrations to support rapid ossification and skeletal development during the early post-hatch period (Costa et al., 2009; El-Katcha et al., 2015). Both studies in our lab emphasize the importance of providing adequate Ca to ensure optimal bone integrity during periods of intense growth, highlighting the necessity for age-specific precision in quail diet formulation (Amoah et al., 2012; Garcia et al., 2024).

Our results indicate that immune response parameters were marginally influenced by dietary Ca levels. The estimated Ca requirement for optimal IgY production was 0.80 %, while Ig-Total required a higher level of 0.852 %. This suggests that immune function, particularly total immunoglobulin production, may require higher dietary Ca levels compared to growth parameters. The relationship between Ca and immune function in poultry has gained increasing attention. A recent research demonstrated that both P and Ca levels significantly influence immune parameters in laying hens (Hofmann et al., 2021). Interestingly, while reduced mineral P enhanced several immune parameters, reduced Ca had the opposite effect, reducing immune function. Their study also revealed strain-specific responses, with certain genetic backgrounds showing different immunological responses to dietary mineral levels (Hofmann et al., 2021). These findings support our observation that immune function may have different mineral requirements than growth parameters. Hofmann et al. (2021) revealed that Ca-deficient diet suppresses T-cell production in laying hens, negatively impacts the immune system. Although the mechanism by which Ca influences immune function is complex and may be differ in different scenarios, the tendency for increased immunoglobulin levels with higher Ca intake observed in our study was aligned with Hofmann et al. (2021) findings that Ca reduction negatively impacts several immune parameters. The higher Ca requirement for Ig-Total (0.852 %) compared to growth parameters (approximately 0.80 %) in our study suggests a potentially higher Ca need for optimal immune function than for growth.

In the present study, a univariate dose–response modeling approach was employed to estimate the dietary Ca requirements of Japanese quail, wherein P levels were held constant across all treatments. This methodological choice was intentional, aiming to isolate the independent effect of dietary Ca on growth performance and bone development, without the confounding influence of Ca × *P* interactions. While it is well recognized that Ca and P have physiological interdependence, univariate models remain widely accepted and practical for establishing the requirement of a single nutrient under controlled conditions. By maintaining a consistent P level, the study avoided additional complexity associated with multivariate factorial designs and enabled precise modeling of Ca impact using various nonlinear regression models. This approach is particularly useful in early-phase studies designed to establish baseline requirements before exploring nutrient interactions in more complex factorial experiments. This comprehensive analysis of multiple response criteria—growth performance, carcass traits, bone characteristics, serum biochemistry, and immune parameters—provides enhanced insight into Ca requirements during the early growth phase of Japanese quails. The consistency in estimated requirements across different growth parameters (gain: 0.788 %, FCR: 0.80 %) and physiological markers (ALP: 0.791 %, IGF: 0.80 %) strengthens the reliability of our findings. However, the variation in estimated requirements between growth parameters (approximately 0.80 %) and immune function (Ig-Total: 0.852 %) highlights the importance of considering the specific production goals when formulating diets for growing quail. Pourmollaei et al. (2024) similarly reported variations in Ca requirements across different parameters, with bone density measures requiring higher Ca levels (0.83-0.87 %) than growth performance (0.75-0.84 %). This differentiation between requirements for various physiological functions demonstrates the complexity of establishing singular nutrient recommendations. Our findings and those of Pourmollaei et al. (2024) challenge the traditional approach of establishing a single nutrient requirement value, instead suggesting that requirements should be defined as ranges specific to different physiological functions. This nuanced understanding enables more precise nutritional management tailored to specific production objectives, whether prioritizing growth, skeletal integrity, or immune function. In the present study, the discrepancy between Ca requirements for growth (0.80 %), Seedor index (0.835 %) and immune function (0.852 %) raises interesting questions about nutritional strategies for different production scenarios, especially in the early growth period. In conventional production settings, formulating diets with 0.80 % Ca may be economically optimal for maximizing growth efficiency. Nonetheless, in systems characterized by elevated disease prevalence, skeletal disorders, or a heightened emphasis on immune resilience, modestly elevated calcium concentrations may be appropriate.

This study demonstrates that dietary Ca levels influence growth performance, carcass characteristics, and bone density in growing quail. The consistency in estimated requirements across different growth criteria strengthens the validity of our findings. The observation that immune function (0.852 %) and bone density (0.835 %) may require higher Ca levels than growth parameters provide new insights into the multifaceted role of Ca in quail physiology (Table 12).

**Table 12:** The best estimation of calcium requirements for different bird responses based on highest R^2^ and lowest error indices (standard deviation of the residuals: *S*_y.x_ and standard deviation: SD).

Response	Requirement	SE^1^	Confidence intervals (CI)	
			95 % Lower CI	95 % Upper CI
Gain	0.788	0.063	0.665	0.911
Feed conversion ratio	0.800	0.114	0.576	1.024
Breast meat yield	0.809	0.018	0.774	0.844
Thigh meat yield	0.748	0.049	0.652	0.844
Seedor index	**0.835**	0.004	0.827	0.843
IgY	0.800	-	-	-
Ig-Total	0.852	0.042	0.770	0.934
Bursa	0.774	0.020	0.735	0.813
Alkaline phosphatase (ALP)	0.791	0.039	0.715	0.867
Insulin-like growth factors (IGF)	0.800	0.069	0.665	0.935

1 Standard error.

## Declaration of competing interest

The authors declare that they have no known competing financial interests or personal relationships that could have appeared to influence the work reported in this paper.
